# Healthcare Service Utilization for Practicing Physicians: A Population-Based Study

**DOI:** 10.1371/journal.pone.0130690

**Published:** 2016-01-11

**Authors:** Yu-Lung Chiu, Senyong Kao, Herng-Ching Lin, Ming-Chieh Tsai, Cha-Ze Lee

**Affiliations:** 1 Graduate Institute of Medical Science, National Defense Medical Center, Taipei, Taiwan; 2 School of Public Health, National Defense Medical Center, Taipei, Taiwan; 3 Sleep Research Center, Taipei Medical University Hospital, Taipei, Taiwan; 4 School of Health Care Administration, Taipei Medical University, Taipei, Taiwan; 5 Division of Gastroenterology, Department of Internal Medicine, Cathay General Hospital, Taipei, Taiwan; 6 Department of Internal Medicine, National Taiwan University Hospital, Taipei, Taiwan; University of Milan, ITALY

## Abstract

**Background:**

Physicians are considered to be the most informed consumers in the use of medical services since they have more information about diseases or medical technology. However, although plenty of researchers have suggested that different medical seeking behavior exists among physicians, very few empirical studies have been conducted to investigate differences in medical utilization between physicians and the general population.

**Objective:**

We explored differences in the utilization of healthcare services between physicians and the general population using a population-based dataset.

**Design:**

A cross-sectional study.

**Participants:**

Data for this study were sourced from the Taiwan Longitudinal Health Insurance Database 2000. We included 1426 physicians and 1426 sex- and age-matched comparison subjects.

**Methods:**

We used Wilcoxon-Mann-Whitney tests to explore differences in variables of healthcare resource utilization between physicians and comparison subjects. We further used Kruskal-Wallis tests to examine differences in variables of healthcare resource utilization between physician practice location and comparison subjects.

**Results:**

We found that physicians had significantly fewer outpatient visits (13.2 vs. 15.7, *p*<0.001) and significantly lower outpatient costs (US$477 vs. US$680, *p*<0.001) than comparison subjects. Furthermore, physicians had lower total health service costs than comparison subjects (US$643 vs. US$1066, *p*<0.001). This indicates that the mean total health service costs in the year 2010 was 1.66-fold greater for comparison subjects than for physicians. We also found that there were significant differences in the mean number of outpatient services (*p*<0.001), outpatient costs (*p* = 0.001), inpatients costs (*p* = 0.018), and total costs (*p* = 0.001) among office-based physicians, hospital-based physicians, and comparison subjects. Specifically, Scheffe contrast tests showed that office-based physicians had significantly more outpatient visits (19.3 vs.10.7, *p*<0.001) and significantly higher outpatient costs (US$656 vs. US$402, *p*<0.001) than hospital-based physicians.

**Conclusions:**

Physicians had less healthcare utilization than comparison subjects. Furthermore, hospital-based physicians had higher inpatient costs and less outpatient services and costs than office-based physicians.

## Introduction

Physicians are considered to be the most informed consumers in the use of medical services since they have more information about diseases or medical technology. Prior studies reported that female doctors had a higher prevalence of use of hormone replacement therapy than other women [1,2]. Another study by Dugowson & Holland also revealed that compared to non-physician families, physician families used more amniocentesis and had a higher rate of primary cesarean sections [3]. However, very few studies have attempted to explore issues of medical utilization behavior of physicians.

Christie & Ingstad indicated that when physicians are ill, they often have difficulty acting as patients [4]. Many studies also consistently found that instead of seeking care, many physicians perform self-treatment when they are sick [4–8]. Two studies even reported that office-based physicians were more likely to practice self-treatment than hospital-based physicians [7,8]. However, although plenty of researchers have suggested different medical seeking behaviors among physicians, very few empirical studies have been conducted to investigate differences in medical utilization between physicians and the general population. According to our knowledge, only one study by Lin et al. reported that physicians were at lower risk of hospitalization than the general population in Taiwan [9]. The literature on medical utilization by physicians is still very limited.

Therefore, in this study, we explored differences in the utilization of healthcare services between physicians and the general population using a population-based dataset. In addition, this study further compared differences in the utilization of healthcare services between hospital-based physicians and office-based physicians.

## Materials and Methods

### Database

Data on the health service utilization of sampled subjects used in this study were retrieved from the Longitudinal Health Insurance Database (LHID2000). The LHID2000 includes sociodemographic information and data on medical claims for 1 million enrollees since initiation of the Taiwanese National Health Insurance (NHI) program in 1995. These 1 million enrollees were randomly selected from all enrollees listed in the 2000 Registry of Beneficiaries (*n* = 23.72 million) under the NHI program by the Taiwan National Health Research Institute. The LHID2000, which was open to the researchers in Taiwan, was available from the National Health Insurance Institute (http://nhird.nhri.org.tw/date_01.html). Hundred studies have been published in international peer-reviewed journals utilizing data from the NHI program [10].

This study was exempt from full review by the Institutional Review Board of the National Defense Medical Center because the LHID2000 consists of de-identified secondary data released to the public for research purposes.

### Study samples

This cross-sectional study consisted of a study group and a comparison group. To select the study group, we first identified 1508 physicians who were practicing medicine in January 1~December 31, 2010. In order to better reflect the actual scenario of physicians practicing in Taiwan, physicians aged over 80 years and under 25 years (*n* = 79) were excluded from the study. We also excluded those who died in the years 2010 and 2011 (*n* = 3) in order to assure equal follow-up periods for all selected physicians. Ultimately, we were left with 1426 physicians who met the study criteria. Of these physicians, 420 (29.5%) and 1006 (70.5%) were office-based and hospital-based physicians, respectively.

We likewise retrieved a comparison group from the LHID2000. We first excluded all subjects aged over 80 years and under 25 years in the year of 2010. We then selected 1426 comparison subjects (1 comparison subject per physician) from the remaining enrollees matched with study subjects by sex and age group (25~39, 40~49, 50~59, 60~69, and >69 years) with the SAS SURVEYSELECT Procedure program (SAS Institute, Cary NC, USA). As a result, this study included 2852 study subjects including 1426 practicing physicians and 1426 matched comparison subjects.

### Variables of interest

The following key variables of healthcare resource utilization were selected: numbers of outpatient visits, inpatient days, and costs of outpatient and inpatient treatment in the year 2010. Taiwan’s NHI is characterized by universal coverage, a single-payer payment system with the government as the sole insurer, comprehensive benefits, access to any medical institution of a patient’s choice, and a wide variety of providers well distributed throughout the country. Therefore, the costs of healthcare services include the costs of diagnoses, medications, treatments, laboratory tests, diagnostic imaging, and surgery.

### Statistical analysis

The SAS statistical package (SAS System for Windows, vers. 8.2; Cary NC, USA) was used for the statistical analyses in the dataset of this study (S1 File). Descriptive statistical analyses, including the frequency, percentage, mean, and standard deviation (SD), were performed on all of the identified variables of healthcare resource utilization. We used Wilcoxon-Mann-Whitney tests to explore differences in variables of healthcare resource utilization between physicians and comparison subjects. We further used Kruskal-Wallis tests to examine differences in variables of healthcare resource utilization between physician practice location and comparison subjects. Differences were considered significant for two-sided *p* values of ≤0.05.

## Results

Table 1 summarizes the descriptive statistics of the sociodemographic characteristics of the sample. The mean age was 44.5 ± 12.1 years of the 2852 study subjects, and were 44.4 ± 12.0 and 44.5 ± 11.9 years for physicians and comparison subjects, respectively (*p* = 0.711). After matching for gender and age group, there were significant differences in monthly incomes (*p*<0.001) between physicians and comparison subjects. Physicians were more likely to have a monthly income of ≥US$830 than were comparison subjects.

**Table 1:** Demographic characteristics of physicians and comparison subjects (*n* = 2852).

Variable	Physicians (*n* = 1426)		Comparison subjects (*n* = 1426)		*p* value
	Total no.	Percent (%)	Total no.	Percent (%)	
Male	1174	82.3	1174	82.3	>0.999
Age (years)					>0.999
25~39	547	38.4	547	38.4	
40~49	411	28.8	411	28.8	
50~59	304	21.3	304	21.3	
60–69	126	8.8	126	8.8	
>69	38	2.7	38	2.7	
Monthly income (US$)					<0.001
$1~530	21	1.5	351	24.6	
$530~830	27	1.9	456	32.0	
≥$830	1378	96.6	619	43.4	
Geographic region					0.151
Northern	667	46.8	706	49.5	
Central	323	22.6	328	23.0	
Southern	406	28.5	355	24.9	
Eastern	30	2.1	37	2.6	

Table 2 presents the use and costs of healthcare services in the year of 2010 for physicians and comparison subjects. Wilcoxon-Mann-Whitney tests revealed that physicians had significantly fewer outpatient visits (13.2 vs. 15.7, *p*<0.001) and significantly lower outpatient costs (US$477 vs. US$680, *p*<0.001) than comparison subjects. Furthermore, physicians had fewer inpatient days (0.713 vs. 1.727, *p*<0.001) and lower inpatients costs (US$167 vs. US$386, *p* = 0.007) compared to comparison subjects. Correspondingly, physicians had lower total health service costs than comparison subjects (US$643 vs. US$1066, *p*<0.001). This indicates that the mean total health service costs in the year 2010 was1.66-fold greater for comparison subjects than physicians.

**Table 2:** Use and costs (US$) of healthcare services in the year 2010 by physicians and comparison subjects.

Variable	Physicians (*n* = 1426)		Comparison subjects (*n* = 1426)		*p* value
	Mean	SD	Mean	SD	
All health services					
Outpatients services (no.)	13.2	12.5	16.6	15.7	<0.001
Outpatient costs (US$)	477	1563	680	2258	<0.001
Inpatient days (no.)	0.713	7.39	1.727	15.789	<0.001
Inpatient costs (US$)	167	1529	386	2676	0.007
Total costs (US$)	643	2381	1066	3770	<0.001

SD, standard deviation.

The use and costs of all healthcare services among office-based physicians, hospital-based physicians, and comparison subjects are presented in Table 3. Kruskal-Wallis tests indicated the existence of significant differences in the mean number of outpatient services (*p*<0.001), outpatient costs (*p* = 0.001), inpatients costs (*p* = 0.018), and total costs (*p* = 0.001) among office-based physicians, hospital-based physicians, and comparison subjects. Specifically, Scheffe contrast tests showed that office-based physicians had significantly more outpatient visits (19.3 vs.10.7, *p*<0.001) and significantly higher outpatient costs (US$656 vs. US$402, *p*<0.001) than hospital-based physicians. In other words, the mean number of outpatient visits was about 1.8-fold higher for office-based physicians than hospital-based physicians. However, differences in inpatient costs and total costs between office-based physicians and hospital-based physicians did not reach a significant level according to Scheffe contrasts tests.

**Table 3:** Use and costs (US$) of healthcare services in the year 2010 by office-based physicians, hospital-based physicians, and comparison subjects.

Variable	Office-based physicians (*n* = 420)		Hospital-based physicians (*n* = 1006)		Comparison subjects (*n* = 1426)		*p* value
	Mean	SD	Mean	SD	Mean	SD	
All health services							
Outpatients services (no.)	19.3	16.0	10.7	11.1	16.6	16.4	<0.001
Outpatient costs (US$)	656	2641	402	733	680	2258	0.001
Inpatient days	0.36	3.85	0.86	8.44	1.73	15.8	0.071
Inpatient costs (US$)	86	690	200	1764	386	2676	0.018
Total costs (US$)	743	2795	602	2184	1066	3770	0.001

SD, standard deviation.

## Discussion

Based on our knowledge, this is the first population-based study to compare utilization of healthcare services between physicians and the general population. We found that physicians consistently had lower outpatient and inpatient services and costs compared to the general population.

Our findings are in line with previous observations that physicians may perform self-diagnosis and treatment instead of seeking medical assistance from other health professionals [11–13]. In addition, Schneck outlined a number of possible factors influencing how physicians respond to illness [14]. These includes a fear of illness as equated to weakness, an inability to reverse roles to become a patient, concerns about loss of confidentiality and privacy, poor choices of personal physicians, and loss of self-esteem when ill. Another study by Steffen also reported that physicians had the following top 3 barriers to receiving healthcare: limited time, concerns about confidentiality, and costs of lost work time [15]. These perhaps explain why physicians have lower medical utilization than the general population.

Furthermore, we found that office-based physicians incurred higher outpatient services and costs than hospital-based physicians. Prior studies indicated that office-based physicians work independently and tend to treat themselves when they have minor health problems [7–8]. Therefore, it is possible that office-based physicians have a greater tendency to practice self-treatment in their own clinics than do hospital-based physicians.

Furthermore, the healthcare system in Taiwan is characterized as a closed-staff system, under which almost all physicians who practice in hospitals are employees of those hospitals, and physicians who practice in clinics do not have privileges at hospitals. Hospital-based physicians are compensated with highly variable combinations of salaries, bonuses, volume-driven fees, and teaching or research supplements. Therefore, their earnings do not fully exhibit a proportional relationship to their clinical efforts. However, incomes of office-based physicians are mainly based on patient volumes, so they have a strong financial incentive to provide more outpatient services including self-treatment in order to reach target incomes [16–18]. This is consistent with our findings that office-based physicians had higher outpatient services and costs than hospital-based physicians.

This study also found that compared to office-based physicians, hospital-based physicians had higher inpatient costs. One study found that hospital-based physicians had a slightly higher risk of hospitalization compared to community practitioners in Taiwan [11]. In contrast to office-based physicians, hospital-based physicians have greater access to inpatient services. This may explain the higher inpatient costs among hospital-based physicians. In addition, a previous study showed that the prevalence of burnout in hospital physicians was significantly higher than in family physicians [19]. Physicians in hospitals are faced with more-complex, more-stressful, and more-irregular patterns of life [20–22]. Burnout is highly prevalent among hospital-based physicians and is accompanied by poor health resulting in high inpatient costs.

A key strength of our study is the use of a population-based dataset to investigate differences in the use of medical utilization among hospital-based physicians, office-based physicians, and the general population in Taiwan. This feature afforded sufficient statistical power and an adequate sample size to detect differences in inpatient and outpatient services among the three groups after adjusting for confounders.

Nevertheless, there are several limitations of this study. First, the LHID2000 used in this study provides no information on working characteristics, lifestyles, health habits, or the disease severity of subjects. Second, most subjects included in our study were of Chinese ethnicity, so the ability to generalize the results to other ethnic groups is uncertain. Finally, this study only employed 1 year of data of healthcare utilization, and this might not fully represent long-term treatment-seeking behavior of the sampled subjects.

Despite these limitations, our population-based study found that physicians had less healthcare utilization than comparison subjects. Furthermore, hospital-based physicians had higher inpatient costs and less outpatient services and costs than office-based physicians. Further large-scale long-period epidemiological studies are suggested to explore differences in the utilization of healthcare services between physicians and the general population in other regions and countries.
